# Supplementary material for: The microbiome biomarkers of pregnant women’s vaginal area predict preterm prelabor rupture in Western China
Source: Front Cell Infect Microbiol. 2024 Oct 31;14:1471027. doi: 10.3389/fcimb.2024.1471027 (PMC11560878; doi:10.3389/fcimb.2024.1471027)
Supplement: Supplementary file 1 [file DataSheet1.zip › compare_1/Community/Structure_Barplot/family/family.taxon.Barplot.Legend.With.superkingdom.pdf]

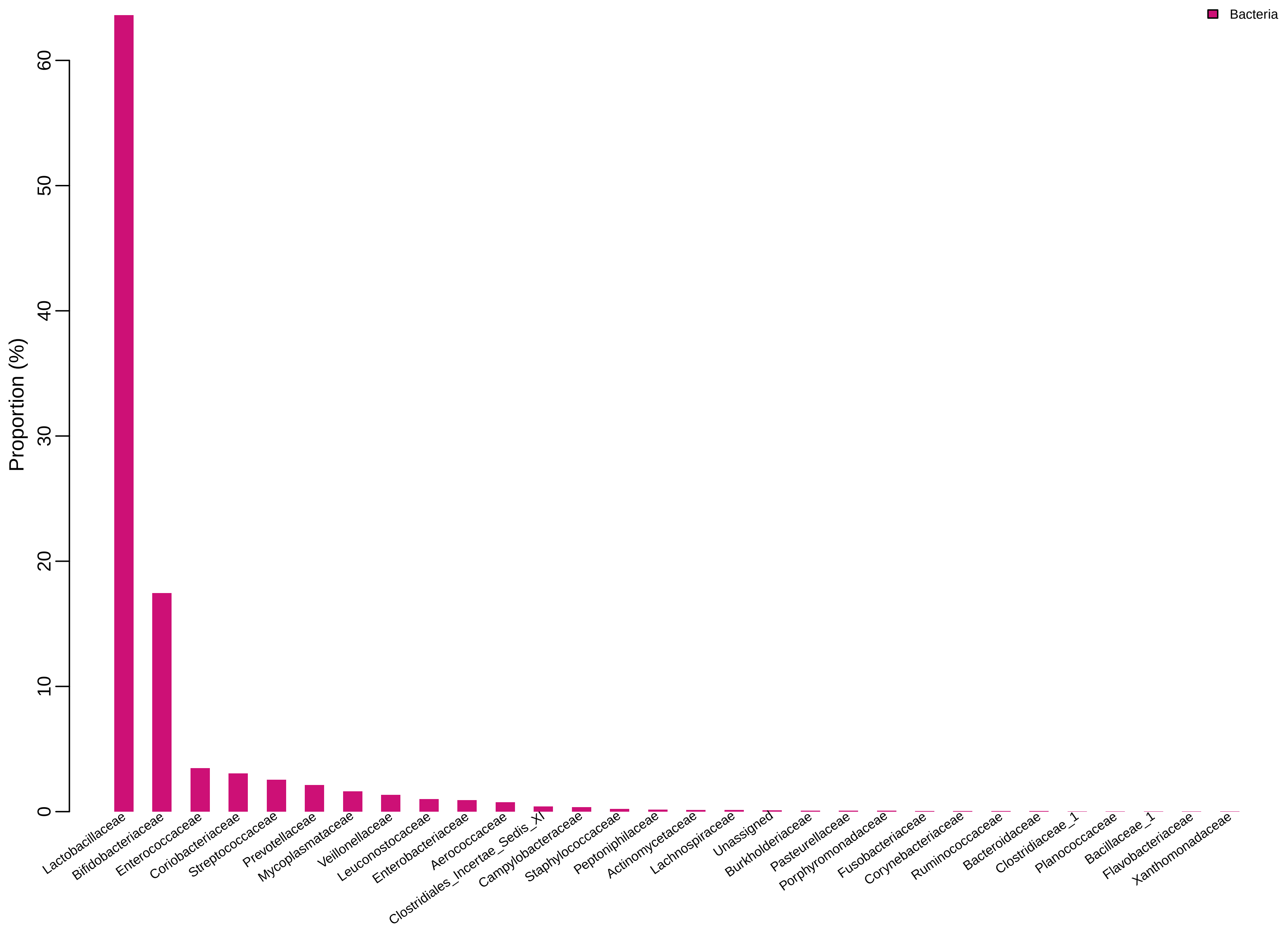
Bacteria
Proportion (%)
0
10
20
30
40
50
60
Lactobacillaceae
Bifidobacteriaceae
Enterococcaceae
Coriobacteriaceae
Streptococcaceae
Prevotellaceae
Mycoplasmataceae
Veillonellaceae
Leuconostocaceae
Enterobacteriaceae
Aerococcaceae
Clostridiales_Incertae_Sedis_XI
Campylobacteraceae
Staphylococcaceae
Peptoniphilaceae
Actinomycetaceae
Lachnospiraceae
Unassigned
Burkholderiaceae
Pasteurellaceae
Porphyromonadaceae
Fusobacteriaceae
Corynebacteriaceae
Ruminococcaceae
Bacteroidaceae
Clostridiaceae_1
Planococcaceae
Bacillaceae_1
Flavobacteriaceae
Xanthomonadaceae
